# Controlled Delivery of Bile Acids to the Colon

**DOI:** 10.14309/ctg.0000000000000229

**Published:** 2020-12-01

**Authors:** Christoph Steiger, Nhi V. Phan, Haoying Sun, Hen-Wei Huang, Kaitlyn Hess, Aaron Lopes, Joshua Korzenik, Robert Langer, Giovanni Traverso

**Affiliations:** 1 Koch Institute for Integrative Cancer Research, Massachusetts Institute of Technology, Cambridge, Massachusetts, USA;; 2 Division of Gastroenterology, Brigham and Women's Hospital, Harvard Medical School, Boston, Massachusetts, USA;; 3 Department of Mechanical Engineering, Massachusetts Institute of Technology, Cambridge, Massachusetts, USA.

## Abstract

**INTRODUCTION:**

Bile acids, such as chenodeoxycholic acid, play an important role in digestion but are also involved in intestinal motility, fluid homeostasis, and humoral activity. Colonic delivery of sodium chenodeoxycholate (CDC) has demonstrated clinical efficacy in treating irritable bowel syndrome with constipation but was associated with a high frequency of abdominal pain. We hypothesized that these adverse effects were triggered by local super-physiological CDC levels caused by an unfavorable pharmacokinetic profile of the delayed release formulation.

**METHODS:**

We developed novel release matrix systems based on hydroxypropyl methylcellulose (HPMC) for sustained release of CDC. These included standard HPMC formulations as well as bi-layered formulations to account for potential delivery failures due to low colonic fluid in constipated patients. We evaluated CDC release profiles in silico (pharmacokinetic modeling), in vitro and in vivo in swine (pharmacokinetics, rectal manometry).

**RESULTS:**

For the delayed release formulation in vitro release studies demonstrated pH triggered dose dumping which was associated with giant colonic contractions in vivo. Release from the bi-layered HPMC systems provided controlled release of CDC while minimizing the frequency of giant contractions and providing enhanced exposure as compared to standard HPMC formulations in vivo.

**DISCUSSION:**

Bi-phasic CDC release could help treat constipation while mitigating abdominal pain observed in previous clinical trials. Further studies are necessary to demonstrate the therapeutic potential of these systems in humans.

## INTRODUCTION

**Figure 1.**
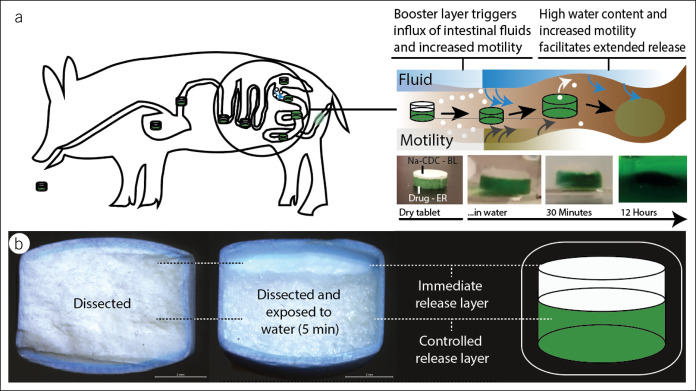
BA delivery system. The delivery system comprises 2 layers. (**a**) After disintegration of the Eudragit S100 coating, the system first releases a small dose immediately. This dose establishes a prosecretory and prokinetic BA level around the delivery system, facilitating further release from the controlled release layer. (**b**) The comparison of a (i) dissected pill and a (ii) dissected pill which was exposed to water for 5 minutes is shown to demonstrate the biphasic kinetic profile of this delivery approach. BA, bile acid.

Bile acids (BAs) facilitate digestion of dietary fats. Increasing evidence suggests that they also play an important role in intestinal motility, humoral activity, and fluid homeostasis. For example, BAs have evolved as pivotal signaling molecules to control gastrointestinal (GI) motility and incretin-mediated metabolomic activity through nuclear hormone farnesoid X receptors and Takeda G protein receptor 5 (1,2). BAs also mediate fluid homeostasis by altering intestinal permeability, electrolyte channels, and expression of aquaporins (3,4). Observational studies linked high fecal BA levels to symptoms of diarrhea such as in patients suffering from irritable bowel syndrome with diarrhea (5,6). Conversely, diseases associated with irritable bowel syndrome with constipation (IBS-C) have been linked to low fecal BA levels (7). Altered BA composition is another factor that can contribute to disease states: In patients suffering from IBS-C, nonsecretory BAs (lithocholic acid) are increased, whereas secretory BAs (chenodeoxycholic acid, [CDCA]) are decreased as compared to healthy controls (6,7). Although a relatively small proportion of individuals have a BA deficiency, the prosecretory and prokinetic properties might be beneficial for to a broad population of patients suffering from constipation (3). Interventional studies delivering secretory BAs such as CDCA, used for the resolution of bile stones, have shown to increase GI motility and loosen stool consistency (8). In female patients suffering from IBS-C, 500–1,000 mg sodium chenodeoxycholate (CDC), delivered as a Eudragit S100-coated capsule formulation, was effective in treating symptoms of IBS-C (9). Among other end points, CDC accelerated colonic transit, increased stool frequency, and loosened stool consistency compared with the control group receiving no treatment. However, approximately half of the patients reported abdominal pain and cramping (0% with placebo, 45% with 500 mg CDC, and 42% with 1,000 mg CDC), and these challenges halted further clinical development. Importantly, abdominal pain also is a key symptom of IBS-C, and this was drastically aggravated by the CDC formulation. Eudragit S100 disintegrates typically in a pH dependent manner in the ascending colon, thereby exposing the drug to GI fluids (10). Given CDC's high solubility and tendency to form micelles, we hypothesized that after disintegration, local “superphysiological” concentrations of CDC trigger adverse effects in the proximal colon. Superphysiological BA concentrations have previously been linked to giant contractions (11) and inflammation (12) in the GI tract. We consequently sought to develop a drug delivery system capable of controlled delivery of CDC to the colon. Conventional colonic drug delivery systems, such as the single-layered release system used as control here within, face the challenge that the decreasing water content along the colon impairs drug dissolution and solubility (13). Given the low free fluid volume in the colon (∼13 in healthy individuals (13)), we sought to develop a bilayered delivery system for the colon that first achieves a prosecretory local concentration of CDC (∼ 5 mM (14)) to facilitate controlled release along the colon from a second layer thereafter (Figure 1). Previous examples of bilayered systems have been applied for release elsewhere in the GI tract for the sequential release of drugs (e.g., Ecofenac CR; Sandoz—diclofenac).


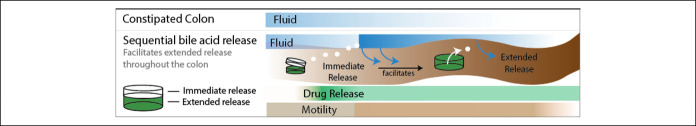


## MATERIAL AND METHODS

### Materials

CDC was acquired from Santa Cruz Biotechnology (Dallas, TX). Eudragit S100 and Aerosil 200 Pharma were acquired from Evonik (Darmstadt, Germany). Magnesium stearate and hydroxypropyl methyl cellulose (HPMC; Mn ∼120,000), acetone, acetonitrile, ammonium acetate, ammonium formate, formic acid, cholic acid, and isopropanol were acquired from Sigma-Aldrich (St. Louis, MO).

### Drug product

One gram CDC gelatin capsules were prepared as previously described with modifications (15,16). In brief, 1 g CDC was filled into 000 sized gelatin capsules from Electron Microscopy Sciences (Hatfield, PA). The capsules were dip coated 8 times with a solution of 8 g Eudragit S100 in 100 mL acetone.

Bilayer systems (10 mm diameter) were compressed using a NP-RD10A tablet press from Natoli Engineering (Saint Charles, MO, Force: 20,000 N). Each pill contained an immediate release layer of 100 mg of CDC per layer and an extended release layer of 400 mg CDC per layer. The immediate release layer was comprised of 97.5% CDC, 2% magnesium stearate, and 0.5% Aerosil 200. The controlled release layer consisted of 87.5% CDC (400 mg), 10% HPMC (Mn∼120,000), 2% magnesium stearate, and 0.5% Aerosil 200. The powder blend was mixed using a FlackTek SpeedMixer (Landrum, SC) at 1,500 revolutions per minute (rpm) for 30 s.

Manufacturing of single layer systems was similar with slight modifications. Each pill was composed of a homogenous layer that contained 87.5% CDC, 10% HPMC, 2% magnesium stearate, and 0.5% Aerosil 200. The average total CDC content per single layer pill was 500 mg.

Bilayer and single layer systems were coated with Eudragit S100 coating following the manufacturer's recommendations (17). Briefly, Eudragit S100 coating solution was prepared by dissolving and homogenizing Eudragit S100, magnesium stearate, and triethyl citrate in a solvent mixture composed of water, acetone. and isopropanol. The tablets were spray-coated for 3 hours with a manual spray nozzle and using an Erweka (Langen, Germany) AR403 pan coater operated at 360 rpm. The tablets where constantly dried using a heat gun operated at 50 °C. The coating solution was pumped through a 1 mm spray nozzle from Glatt (Binzen, Germany) at 3.5 mL/min using a Masterflex L/S 7528-30 peristaltic pump from Cole-Parmer (Vernon Hills, IL). The setup was operated with approximately 150–300 g of 4.5 mm glass plating beads from Zymo Research (Irvine, CA) as filler matter.

### In vitro dissolution & analytics

Dissolution experiments were performed using a Hanson (Chatsworth, CA) Vision Elite 8 dissolution testing system (37 °C, stirred at 100 rpm). The delivery systems were exposed to 800 mL of simulated gastric fluid (without enzymes, United States Pharmacopeia) for 1 hour, before transferring to simulated intestinal fluid for 1 hour (SIF, without enzymes, pH 6.8). After 1 hour, the pH was adjusted to 7.4 (2 M NaOH). In another set of experiments, the CDC capsules and bilayer pills were exposed to SIF (pH 6.8, 1 hour) for 1 hour before they were transferred to SIF (pH 7.4). These experiments were performed at 50 rpm under otherwise identical conditions.

CDCA concentrations were then analyzed using an Agilent (Santa Clara, CA) 1,260 Infinity II HPLC system equipped with an Agilent 6120B mass spectrometer. Data processing and analysis were performed using OpenLab CDS ChemStation (Agilent). Isocratic separation was achieved using an Agilent 4.6 × 50 mm EC C-18 Poroshell column with 2.7 μm particles, maintained at 55 °C. The mobile phase consisted of 20% 10 mM ammonium acetate in water (unbuffered) and 80% methanol using a flow rate of 0.850 mL/min over a period of 6 minutes. Gradient separation was achieved over a 5 minutes run time (3 minutes post run). The injection volume was 5 μL, and the selected ultraviolet detection wavelength was 210 nm at an acquisition rate of 5 Hz. The electrospray ionization drying gas flow rate and temperature was 10 L/min and 350 °C, accordingly. The single ion monitoring of CDCA and cholic acid internal standard were analyzed using negative mode electron spray ionization at a gain of 2.00, fragmentor of 70, monitoring mass-to-charge ratios of 391.40 m/z and 408.60 m/z, respectively.

### Pharmacokinetic simulation

**Figure 2.**
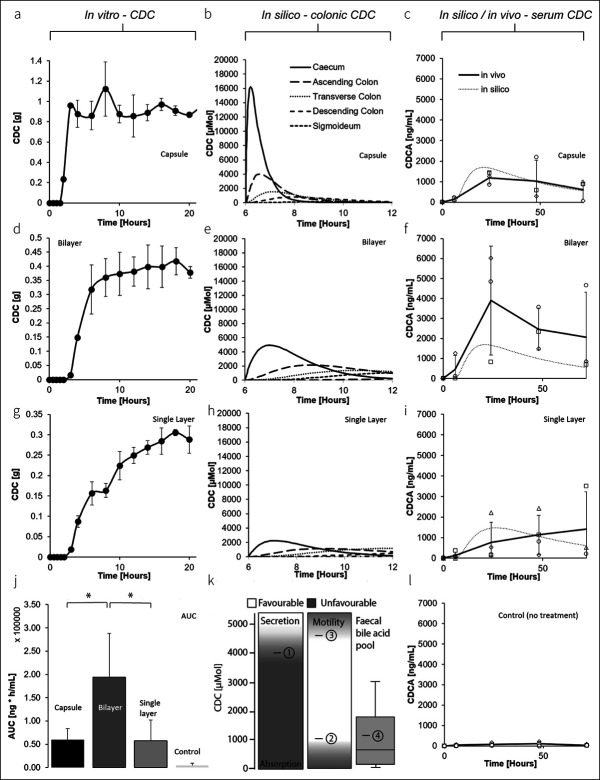
Pharmacokinetic evaluation of the bilayer delivery system vs traditional formulations. *In vitro* release pattern of (**a**) 1 g CDC capsules (used in a clinical front-runner study (9) linked to a high frequency of abdominal pain), (**d**) bilayered delivery systems, and (**g**) single-layered delivery systems (serving as control for a conventional extended release delivery system exposed to SGF [1 hour], SIF [0.2M, SIF, pH 6.8, 1 hour], and SIF [0.2M, pH 7.4], respectively [100 rpm]). An in silico pharmacokinetic model was used to understand the CDC levels over time in the colon for (**b**) 1 g CDC capsule, (**e**) the bilayered delivery system (×2), and the (**h**) single-layered delivery system (×2). For comparison, (**k**) colonic CDC levels that have previously been linked to secretory (1) (14), prokinetic (2) (18), and distinct prokinetic (3) (19) effects are shown in comparison to phycological bile acid levels (4) (6). The pharmacokinetic model was also used to predict systemic bile acid levels in swine (dashed line), which are shown in comparison to systemic levels (solid lines, LC-MS) for (**c**) 1 g CDC capsules, (**f**) bilayered delivery systems (×2), and (**i**) a single-layered delivery system (×2, n = 4). The results are depicted as the mean of n = 3 ± SD unless otherwise noted (*P ≤ 0.05). CDC, sodium chenodeoxycholate; SGF, simulated gastric fluid; SIF, simulated intestinal fluid.

We used Symbiology from MathWorks (Natick, MA) to generate a 6-compartment GI model (see Figure 2, Supplementary Digital Content 1, http://links.lww.com/CTG/A400 for setup). The compartments modeled were the caecum, ascending colon, transverse colon, descending colon, and sigmoid. We assumed first order kinetics for all reactions (for all parameters used in calculations, please see Table 1, Supplementary Digital Content 1, http://links.lww.com/CTG/A400). For parametric data, a one-way analysis of variance after a post hoc Duncan test was used. A *P* ≤ 0.05 was considered statistically significant.

### In vivo porcine studies

All procedures were conducted in accordance with the protocols approved by the Massachusetts Institute of Technology Committee on Animal Care and as previously described with minor modifications. Twelve separate female Yorkshire pigs weighing approximately 30–50 kg were randomly assigned for *in vivo* evaluation. Female pigs were used because they are less aggressive and more successfully socially housed in compatible groups. Investigators were not blinded. After overnight fasting, the animals were sedated with Telazol (tiletamine/zolazepam) 5 mg/kg, xylazine 2 mg/kg, and atropine 0.04 mg/kg. Endotracheal intubation was performed, and anesthesia was maintained with isoflurane thereafter (1%–3% in oxygen). For pharmacokinetic studies, the delivery systems were placed into the small intestine by using an esophageal overtube (US Endoscopy, Mentor, OH) with endoscopic guidance. Blood was sampled through a central venous catheter or through mammal bleeds in case catheter placement was not possible.

Disintegration of bilayer systems *in vivo* was profiled in a terminal procedure in swine. After sedation and intubation with isoflurane, a laparotomy was performed using a ventral middle line incision from the xiphoid to the pubis. The delivery systems were then placed through an incision into the duodenum and retrieved 4 hours later. A set of unrelated different experiments were performed concurrently during the terminal procedure but did not interfere with the GI tract either physically nor pharmacologically.

### Rectal manometry

We analyzed the effect of BA formulations on rectal motility. After sedation (see above), the rectum was cleaned with ∼ 120 mL saline infused through a Foley catheter. One uncoated CDC capsule or one uncoated bilayer delivery system was applied rectally along with 50 mL saline before measurement was started. No CDC was applied in the control group. The measurement was performed by the rectal placement of an air-inflated balloon connected (polyvinyl chloride tube) to a micro pressure sensor (MPRLS; Honeywell Charlotte, NC). The balloon was inflated with a 50 mL syringe to ensure comparability among the groups. The sampling frequency of the was 30 Hz. Data processing were performed in an unblinded fashion on MATLAB (Mathworks, Natick, MA) using an ultralow band pass finite impulse response filter with cutoff frequency of 0.1 Hz to filter auxiliary signals (respiration) and identify rectal motility.

### In vivo analytics

CDCA in serum from *in vivo* experiments were analyzed using ultra-performance liquid chromatography-tandem mass spectrometry. Analysis was performed on a Waters ACQUITY UPLC-I-Class System aligned with a Waters Xevo TQ-S mass spectrometer (Waters Corporation, Milford MA). Liquid chromatographic separation was performed on an Acquity UPLC HSS T3 (50 × 2.1 mm, 1.8 μm particle size) column at 50 °C. The mobile phase consisted of aqueous 0.1% formic acid, 10 mM ammonium formate solution (mobile phase A), and acetonitrile: 10 mM ammonium formate, 0.1% formic acid solution (95:5 v/v) (mobile phase B). The mobile phase had a continuous flow rate of 0.6 mL/min using a time and solvent gradient composition.

For the analysis of CDCA, the initial composition, 95% mobile phase A, was held for 1.00 minutes, after which the composition was changed linearly to 2% mobile phase A until 1.80 minutes. The composition of 2% mobile phase A and 98% mobile phase B was held constantly until 3.00 minutes. The composition linearly changed to 80% mobile phase A at 3.50 minutes and was linearly returned to 95% mobile phase A until completion of the run, ending at 4.50 minutes, where it remained for column equilibration. The total run time was 4.50 minutes. The mass to charge transitions (m/z) used to quantitate CDCA was 391.452 > 391.591. For internal standard, d-4 CDCA, 395.38 > 396.07 m/z transition was used for quantitation.

Sample introduction and ionization was by electrospray ionization in the positive ionization mode. Waters MassLynx 4.1 software was used for data acquisition and analysis. Stock solutions were prepared in methanol at a concentration of 500 μg/mL. A 12-point calibration curve was prepared in analyte-free, blank serum ranging from 1.25 to 5,000 ng/mL. One hundred microliter of each serum sample was spiked with 200 μL of 250 ng/mL internal standard in acetonitrile to elicit protein precipitation. Samples were vortexed, sonicated for 10 minutes, and centrifuged for 10 minutes at 13,000 rpm. Two hundred micro liter of supernatant was pipetted into a 96-well plate containing 200 μL of water. Finally, 10.00 μL was injected onto the UPLC-ESI-MS system for analysis. One serum sample was lost (72 hours, single layered) and we included a fourth animal for this group.

## RESULTS

### In vitro release

After pH-triggered disintegration of Eudragit S100, CDC was immediately and completely released with a half-life of 0.5 hours from the capsule formulation (100 rpm, Figure 2a, see Table 1, Supplementary Digital Content 1, http://links.lww.com/CTG/A400; lag time: 1.5 hours). Under identical conditions, the bilayer system released CDC after first order kinetics with a half-life of approximately 2.5 hours (Figure 2d, see Table 1, Supplementary Digital Content 1, http://links.lww.com/CTG/A400; lag time: 2 hours). The single layer system, serving as a control for a conventional colonic drug delivery system with sustained release kinetics, released CDC with a half-life of approximately 6 hours (Figure 2g; lag time: 2 hours).

**Figure 3.**
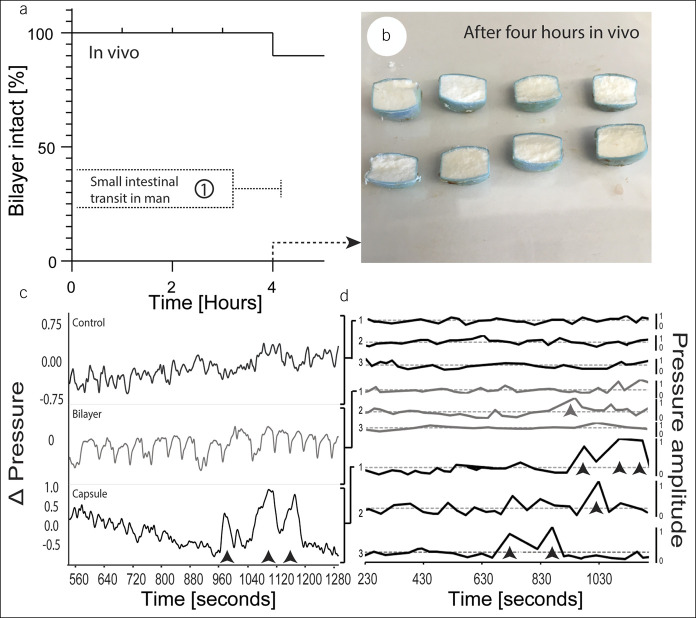
*In vivo* evaluation of CDC bilayer delivery systems as compared to the capsule formulation (used in a clinical frontrunner study and linked to a high frequency of abdominal pain (9)). (**a**) Kaplan-Meier plot demonstrating the bilayer delivery system's capacity to maintain integrity throughout intestinal passage in swine. The results are shown in comparison to small intestinal transit time in man (1) (21). (**b**) Shows retrieved dissected delivery systems after 4 hours. (**c**) Representative rectal manometry patterns after rectal placement of uncoated bilayer delivery systems or CDC capsules. Amplitudes of 3 rectal manometry patterns are shown in (**d**). Giant contractions are indicated by arrowheads (data normalized to the mean amplitude). CDC, sodium chenodeoxycholate.

Importantly, when performing the release experiment at 50 rpm, we observed a biphasic release pattern initially releasing ∼ 150 mg CDC within 3 hours after pH-triggered disintegration (see Figure 3a, Supplementary Digital Content 1, http://links.lww.com/CTG/A400). After first order kinetics, the bilayer tablet released CDC over a course of 16 hours thereafter (∼ 80% CDC released). Under identical conditions (e.g., 50 rpm), the capsule formulation released its full payload within 0.5 hours (see Figure 3b, Supplementary Digital Content 1, http://links.lww.com/CTG/A400).

### Pharmacokinetic model and in vivo pharmacokinetics

To better understand local colonic CDC levels, we generated a 6-compartment pharmacokinetic *in silico* model based on the physiological characteristics and the *in vitro* pharmacokinetic profile of the delivery systems (see Table 1, Supplementary Digital Content 1, http://links.lww.com/CTG/A400). We observed local colonic peak concentrations of 16.2 (6.2 hours), 5.0 (7 hours), and 2.2 mM (7 hours) for the capsule, for the bilayer, and for the single layer formulation, respectively (Figure 2 b, e and h). Modelled systemic CDC levels reached c_max_ after 19.3 hours (1,696 ng/mL), 21.6 hours (1,694.7 ng/mL), and 27.45 hours (1,477 ng/mL), respectively (Figure 2 c, f and i). *In vivo* serum CDCA levels after intestinal placement of the delivery systems matched modelled levels, and c_max_ was 1,182 ± 296.43 (24 hours), 3,895.93 ± 2,725.87 (24 hours), and 1,409.40 ± 1,825.31 (72 hours) for capsule, bilayer, and single layer, respectively (Figure 2 c, f and i). Control experiments were performed in pigs not receiving CDC. Maximal CDCA levels were 87.36 ± 107.63 in this group (Figure 2 c, f and l).

### GI transit

To understand *in vivo* disintegration characteristics of the delivery system, we retrieved the release systems after intestinal passage in swine during a terminal procedure. 91.6% (11 systems) of the release systems placed into the SI (12) remained intact until retrieval 4 hours after implantation (Figure 3; see see Figure 1b, Supplementary Digital Content 1, http://links.lww.com/CTG/A400 for partly disintegrated system).

### Rectal manometry

A clinical challenge of colonic CDC delivery is noticeable abdominal cramping and pain in patients (9). We had hypothesized that this symptom was because of massive contractions triggered by local superphysiological CDC levels within the proximal colon. We hence performed rectal manometry after the placement of uncoated CDC capsules and bilayer systems in comparison to the control receiving no treatment. Approximately 15 minutes after rectal application, we observed repeated massive contractions in all pigs receiving CDC capsules by measuring the pressure variation in the colon (Figure 3c, see Figure 4, Supplementary Digital Content 1, http://links.lww.com/CTG/A400 for primary data). Figure 3d shows the unified and filtered colon pressure variation by means of a low pass filter. In the group receiving the bilayer system, we only observed one major contraction. In the control group, we did not observe any massive contractions. One control measurement (depicted in see Figure 4, Supplementary Digital Content 1, http://links.lww.com/CTG/A400) was excluded because of a loss in pressure.

## DISCUSSION

This study explores novel delivery systems designed to overcome current challenges of colonic BA delivery. Specifically, we investigate the pharmacokinetic challenges of a capsule-based formulation used to demonstrate CDC's therapeutic role as a prokinetic laxative in patients suffering from IBS-C (9). We speculate that adverse effects associated with this experimental treatment were caused by local superphysiological CDC levels because of an unfavorable kinetic profile of the capsule-based formulation. We used data from previous preclinical and clinical BA perfusion studies as a point of reference to better understand the profile required for BA drug delivery systems (Figure 2k): in man, prokinetic effects of CDCA have been described as low as 1 mM (18). Distinct prokinetic and prosecretory effects have been described for 5 mM (∼ 5 times increased motility index) (14,19). Based on this profile, we sought to develop a delivery system that can establish a concentration of ∼ 5 mM throughout the colon. Extended delivery along the colon is impaired because drug delivery systems travel along the colon and are exposed to increasing viscosity and decreasing fluid content (13). We hypothesized that this is particularly relevant in constipated patients and chose to address this with a sequential release approach: the bilayered release system first achieves a prosecretory local concentration of CDC (∼ 5 mM (14)) to facilitate controlled release along the colon from a second layer thereafter (Figure 1). The local concentration of ∼ 5 mM CDC is particularly important to establish if the delivery system is intended to travel to distal parts of the colon before it degrades. A lack of fluid and high viscosity could render controlled release ineffective. As suggested here within, this could be addressed by locally increasing the colonic fluid level with prosecretory drugs such as CDC.

Targeted disintegration of colonic delivery systems in general is challenging, given the high variability of the colonic environment. We have used Eudragit S100, which is approved by global authorities for oral drug delivery. Eudragit S100 is a copolymer composed of poly(methacrylic acid-co-methyl methacrylate). Compared with methacrylic acid copolymers that degrade immediately on encountering the gastroduodenal pH shift (e.g., Eudragit L), Eudragit S100 is composed of more methyl ester moieties, rendering the polymer more hydrophobic. This decreases the solubility such that the coating of a dosage form can degrade steadily over a period of ∼3 hours when the medium pH is raised above ∼ pH 7 (20). Because small intestinal transit time is relatively constant (∼3 ± 1 hour) (21), successful delivery to the distal small intestine or the colon is likely to occur, but delivery failures (such as nondisintegrated delivery systems) occur frequently in humans (10). Combinatorial approaches that leverage microbial enzymatic activity on top of pH shift (e.g., Phloral) are available and could be leveraged for bilayered delivery systems as well to improve targetability (22).

Using *in silico* modelling and based on data from *in vitro* dissolution studies, we estimated a local c_max_ (CDC) for the capsule formulation that exceeds the minimal prokinetic concentration 15 times and exceeds the minimal prosecretory concentration 3 times (∼ 15 mM in the caecum [Figure 2b]). We chose an *in silico* model given the high variability of GI transit and degradation of oral solid dosage forms (13). These factors and the microbial metabolization of CDC make an accurate determination of colonic CDC levels technically complex. We used pharmacokinetic modelling to predict systemic CDC levels based on *in vitro* dissolution profiles. We observed that the modelled systemic pharmacokinetic profile matched the *in vivo* profile for the capsule formulation (Figure 2c). Systemic *CDCA in vivo* levels, however, were higher in the bilayer group as compared to the modelled levels (Figure 2c,j**)**. Absorption kinetics of CDC are inversely correlated with colonic concentrations (23), which explains the low amount of systemic CDC in the capsule group as compared to the bilayer group (Figure 2j). Given similar compositions (80% of the formulation is identical, Table 1), the distinct different pharmacokinetic profile of the bilayer pill compared with the single layer pill is surprising (area under the curve of bilayer: 3.4 times area under the curve of single layer formulation, Figure 2j). This supports our hypothesis that a biphasic release of CDC can be leveraged to increase the amount of CDC being release in the colon. Low colonic fluid content can impair drug dissolution, and this seems to be particularly important for drug delivery systems designed for treating constipation (13).

**Table 1.**
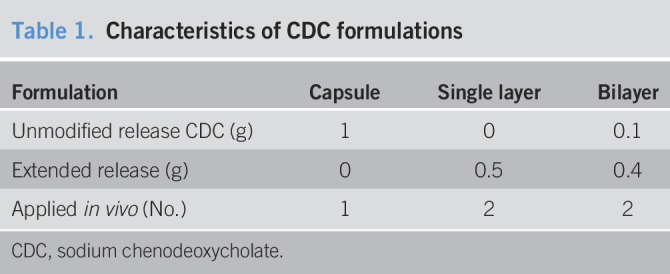
Characteristics of CDC formulations

Formulation	Capsule	Single layer	Bilayer
Unmodified release CDC (g)	1	0	0.1
Extended release (g)	0	0.5	0.4
Applied *in vivo* (No.)	1	2	2

CDC, sodium chenodeoxycholate.

A limitation of this study is the lack of comparative preclinical data demonstrating efficacy and the absence of abdominal pain in a large animal constipation model. We did not perform this study given the complexity to measure pain in large animal models, ethical implications, and a lack of appropriate large animal constipation models. We focused our initial manometric evaluation in the rectum rather than full colonic evaluation, and therefore, future characterization should include further characterization of the entire colon*.* Both the lack of physical movement and focus on the rectum rather than the colon can decrease the clinical significance of this animal model. In addition, we observed large interindividual variability of manometric measurements, which could be minimized by repeated CDC challenge within one animal. Likewise, CDC *in vivo* was applied via intestinal placement because swine generally cannot be trained to voluntarily ingest a whole dosage form. In addition, swine gastric transit is exceedingly slow. Given these 2 challenges, we dosed the animals through direct dosage placement in the duodenum. Future work will include large animal experiments to further understand the time-resolved pharmacodynamic and pharmacokinetic effects of this delivery system. Previous clinical efficacy in IBS-C (9) and the existing regulatory framework on CDCA (24,25) can guide pharmaceutical development for future clinical studies. We intend to demonstrate the therapeutic potential of such an approach in patients suffering from IBS-C.

Controlled delivery of BAs to the colon provides a novel approach to treating GI diseases, such as constipation. Previous studies demonstrated their efficacy in treating IBS-C; however, abdominal cramping occurred in almost half of the study population, hindering further therapeutic application. We linked the fast release kinetics of a clinically used capsule formulation to giant contractions in swine and suggest an extended delivery approach accordingly. We developed a delivery system with biphasic kinetics and profiled its capacity to address delivery failures because of low colonic fluid. A 4-fold increase in the bioavailability of CDC as compared to an identically designed conventional extended formulation release formulation demonstrated increased availability of CDC. Further studies are necessary to demonstrate the therapeutic potential of these systems in humans.

## CONFLICTS OF INTEREST

**Guarantor of the article:** Giovanni Traverso, MB, BChir, PhD.

**Specific author contributions:** Christoph Steiger, Dr. rer. nat. and Nhi V. Phan, MEng, contributed equally to this work. C.S.: planning and conducting the study, collecting and interpreting data, and/or drafting the manuscript. N.V.P.: planning and conducting the study, collecting and interpreting data, and/or drafting the manuscript. H.S.: planning and conducting the study and collecting and interpreting data. H.-W.H. and K.H.: collecting data. A.L.: collecting the data. J.K.: planning and conducting the study. R.L.: supervision and manuscript review. G.T.: study design, supervision, manuscript preparation, and review.

**Financial support:** Alexander von Humboldt foundation (Feodor Lynen Fellowship to C.S.). Department of Mechanical Engineering, Massachusetts Institute of Technology (G.T.).

**Potential competing interests:** C.S., J.K., R.L. and G.T. are coinventors on a patent application describing the bile acid delivery systems described here within. C.S., J.K., R.L. and G.T. have a financial interest in Bilayer Therapeutics, Inc. This company is developing oral bile acid delivery systems.

**Study Highlights**

**WHAT IS KNOWN**

✓ CDC has shown early clinical efficacy in treating IBS-C by inducing accelerated colonic transit and increasing stool frequency.
✓ CDC dosing was associated with a high frequency of intense abdominal pain among patients receiving CDC, preventing further clinical development of this experimental treatment.

**WHAT IS NEW HERE**

✓ We demonstrate that local superphysiological CDC levels caused by colonic bolus delivery of CDC can trigger giant contractions in pigs.
✓ We formulated controlled release matrix formulations and demonstrated in pig the potential to deliver CDC to the colon while mitigating giant contractions.
✓ We demonstrate that potential delivery failures of controlled release matrix formulations because of low colonic fluid can be addressed by using bilayered delivery systems.

**TRANSLATIONAL IMPACT**

✓ Matrix-based CDC formulations could facilitate colonic CDC delivery to treat IBS-C while addressing adverse events which have previously been associated with this experimental therapy.
